# River Otter Predation of Nesting Seabirds Along the Coasts of North America

**DOI:** 10.1002/ece3.72118

**Published:** 2025-09-13

**Authors:** Luke Stuntz, Rachael A. Orben

**Affiliations:** ^1^ Department of Fisheries, Wildlife, and Conservation Sciences Oregon State University Oregon USA; ^2^ Department of Fisheries, Wildlife, and Conservation Sciences, Hatfield Marine Science Center Oregon State University Oregon USA

**Keywords:** coastal ecology, *Lontra canadensis*, predation, river otter, seabird, seabird breeding biology, seabird colonies

## Abstract

River otter ( *Lontra canadensis* ) predation of seabirds has been periodically documented throughout North America, but most knowledge about this subject exists as anecdotal observations in the memories of experienced seabird biologists. Using surveys, interviews, white papers, and published literature, we collected detailed records of seabird predation by river otters at 73 islands on the coasts of North America. From this dataset, we were able to draw conclusions about the seabird species experiencing river otter predation, the geographic distribution of predation records, and factors associated with river otter occurrence at colony islands. River otters preferentially select for small‐bodied burrow‐nesting species, with predation of ground‐nesting species occurring mostly opportunistically. Predation of nesting seabirds occurs across the entire Pacific range of the river otter and throughout the northern Atlantic, but we did not locate a single record of this behavior on the Atlantic coast south of Maine; we hypothesize that this is due to geographic differences in nesting seabird assemblages. The presence of burrow‐nesting seabirds and proximity to the mainland are both strong predictors of river otter occurrence at colony islands. River otters will swim up to eight kilometers across open ocean to access seabird colonies, but predation is significantly more common at nearshore islands (median distance of 1.7 km). Reports of seabird predation by river otters in published literature are rare, are heavily biased toward an ornithological audience, and have decreased over time as “Field Notes” style publications have decreased in popularity. Despite being more widespread than previously reported, predation by river otters appears to be sustainable for most colony nesting seabird populations.

## Graphical abstract

Northern river otter predation of nesting seabirds is rarely reported in published literature, but this behavior is common throughout the Pacific coast and on the northern Atlantic coast. Through surveys of seabird field biologists, we documented records of river otters hunting eighteen different species of nesting seabirds at 73 different colony islands in the United States and Canada. River otters show a selective preference for small‐bodied burrow‐nesting seabird species, with predation of larger surface‐nesters occurring mostly opportunistically.

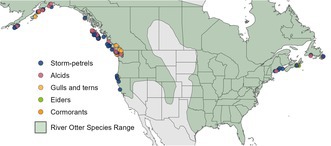

## 1 Introduction

The damaging effects of introduced predators on nesting seabirds at offshore colony islands has led to an implicit assumption in much of the published literature that terrestrial carnivores and colonial seabirds cannot coexist (Courchamp et al. 2003; Hays and Conant 2007; Croxall et al. 2012; Nogales et al. 2013). What this paradigm ignores, however, is that seabirds nesting on nearshore islands and along the coastal mainland are often sympatric with native carnivores. For example, arctic foxes ( *Vulpes lagopus* ) (e.g., Larson 1960; Eide et al. 2005) and polar bears ( *Ursus maritimus* ) (e.g., Stempniewicz 1993; Dey et al. 2020) often overlap in habitat use with nesting seabirds at islands and mainland sites throughout the Arctic, where they will occasionally forage on eggs, chicks, and adult seabirds. Impacts to Arctic‐nesting seabird populations by both of these species seem to be mediated by the abundance of other preferred prey resources and by territoriality between carnivores (Pagh and Hersteinsson 2008; Prop et al. 2015). Some additional observations of predation by smaller‐bodied mustelids suggest that seabird behavioral responses may also help to insulate populations from the impact of terrestrial predation, with these responses most commonly involving nesting site selection (e.g., Barros et al. 2016) and temporary cessation of breeding attempts (e.g., Oro et al. 1999).

In North America, seabirds nesting in the nearshore area often share this environment with a highly adaptable semi‐aquatic carnivore: the northern river otter ( *Lontra canadensis* ). Despite the implications of their common name, river otters readily forage on marine resources, and coastal areas can have higher population densities than freshwater habitats (Blundell et al. 2000). River otters primarily hunt fish and crustaceans, but their dietary composition generally tracks the abundance of available prey, and they have been observed to take large amounts of amphibians, mammals, and birds in specific circumstances (Kruuk 2006). Given this ecological context, it should not be surprising that river otters will occasionally prey upon seabirds. In marine environments, it is expected that rafting seabirds would be similarly vulnerable to predation as waterfowl at inland lakes (Baillie et al. 2004). However, predation on seabird colonies would appear less likely due to the need to cross open stretches of ocean and to hunt on land, which is relatively rare for river otters (Hansen 2003). Regardless, this behavior occurs on both coasts of North America, with some records indicating the predation of hundreds of nesting seabirds from a single colony (e.g., Quinlan 1983; Hoeg et al. 2021).

River otter prey selection and habitat preferences are well studied in freshwater systems (Melquist and Maurice 1983; Hansen 2003; Day et al. 2015), but it is unclear how well this might translate to their ecology at seabird colony islands. To hunt nesting seabirds, river otters must cope with the energetic costs of swimming to colony islands, adapt their foraging to a different pattern of prey availability (highly seasonal and hyper‐abundant), and use very different hunting tactics. River otters generally select for benthic, slow‐moving aquatic prey available in high abundance (Ryder 1955; Cote et al. 2008; Day et al. 2015), suggesting that they rely on a “risk averse” foraging strategy that focuses on consistently catchable prey rather than targeting species that might provide a greater energetic reward per capture (Pyke 1984). Seabird predation would seem to represent a break in this strategy, but risk aversion could result in river otters selecting for certain morphological and behavioral traits in avian prey. River otter habitat selection may also be markedly different at coastal islands—while river otter distribution in inland systems is driven by the availability of prey and preexisting riparian shelter (Melquist and Maurice 1983), topographic variables such as tidal slope steepness and shoreline convexity take on primary importance in coastal areas (Bowyer et al. 2003; Albeke et al. 2010). Habitat selection may be further altered by the unique social patterns of river otters in coastal areas, where they generally form larger groups, occupy smaller home ranges, and exhibit lower territoriality (Blundell et al. 2000, 2004; Barocas et al. 2016, 2021). The addition of seabird colony islands as a highly concentrated foraging resource should be expected to further complicate coastal habitat selection, and the factors which determine site use may be unique to this foraging context.

Seabirds are among the most threatened groups of birds, with anthropogenic impacts resulting in population declines for the majority of species (Croxall et al. 2012; Dias et al. 2019). While invasive alien species are the primary on‐land threat for nesting seabirds, the context of compounding anthropogenic stressors further increases the impacts of native predators (Dias et al. 2019). Almost all seabird species have low reproductive rates and long lifespans (Hamer et al. 2001), and these traits amplify the effects of additive sources of mortality on long‐term population trajectories (Purvis et al. 2000; Sæther and Bakke 2000). Predation at seabird colonies tends to result in Allee effect dynamics, with birds at smaller colonies experiencing higher rates of predation due to a reduced dilution effect, lower collective vigilance, and less effective group anti‐predator defenses (Schippers et al. 2011). As anthropogenic threats cause progressive reductions in seabird colony size, predation can begin to result in an acceleration toward colony collapse or abandonment (McDowall and Lynch 2019). Even in locations where predation does not cause serious reductions in population, the presence of native predators can result in shifts in nest attendance (Corkery et al. 2015; Marsteller et al. 2024), changes to socialization and breeding behavior (Mougeot and Bretagnolle 2000), and costly physiological stress responses (Schaefer and Colombelli‐Négrel 2021; Geldart et al. 2023). While species such as river otters are a natural part of the coastal island ecosystem, understanding their impacts on nesting seabirds is increasingly important for conservation in the context of global anthropogenic change. By collecting anecdotal records of river otter activity at seabird colonies from biologists working throughout North America, we aim to provide insight into the frequency of seabird predation, the species at greatest risk from river otters, and the factors driving river otter occupancy of coastal seabird islands.

The first aim of this paper was to assess differences in predation risk for various seabird taxa; that is, what makes a particular seabird species more vulnerable to river otter predation? We considered the non‐exclusive hypotheses that body size, nest structure (surface vs. crevice vs. earthen burrow), and timing of colony attendance (nocturnal vs. diurnal) would all be associated with predation rates. We predicted that river otters would preferentially select for smaller‐bodied birds, since these may be easier to quickly subdue and kill (similar to how river otters preferentially hunt slower fish). We also predicted that river otters' fusiform morphology, ability to dig, and well‐documented use of earthen burrows for natal dens would seemingly make them naturally adapted predators of burrow‐nesting seabirds. Many burrow‐nesting seabird species exclusively visit colonies at night, and we speculated that these species would be at greater risk for predation from river otters, which are primarily nocturnal hunters (albeit with a good deal of spatial and seasonal variability; Kruuk 2006; Martin et al. 2010).

The second aim of this paper was to understand which spatial factors drive patterns of river otter occurrence at seabird colony islands. We made the a priori hypotheses that decreasing distance to the shore, increasing island size, and the presence of persistent freshwater would all be associated with an increased risk of predation by river otters. While more proximate islands would be easier to access for transitory predation, larger islands and those with persistent freshwater would seemingly be more suitable for long‐term residence by river otters. The presence of freshwater is critical for the maintenance of river otters' coats; river otters foraging in coastal habitats return to freshwater pools on a daily basis to groom (Kruuk 2006). Without a layer of insulating fat, the condition of a river otter's fur is critical for maintaining thermoregulatory capacity in cold water foraging conditions (Tarasoff 1972). We also predicted that the presence of burrow‐nesting seabirds would increase the probability of river otter occurrence, based on our previously stated hypothesis that burrow nesters may be the preferred seabird prey of river otters.

We accomplished these study aims by collecting anecdotal observations of seabird predation from seabird biologists and mammologists throughout North America. Because this project focuses on a terrestrial carnivore at the very edge of its ecological niche, we were interested in how knowledge of this subject would differ between biologists with different taxonomic specializations. We hypothesized that there would be a gap in understanding about this species interaction between seabird biologists and mammologists due to bias in the location of field research and underpublishing of predation records. We expected that river otter biologists would underestimate the risk that otters pose to seabirds, that seabird biologists would rarely publish their observations of predation, and that any publications on the subject would be strongly skewed towards an ornithological audience.

## 2 Methods

### 2.1 Nesting Seabird Prey Selection by River Otters

To collect anecdotal records of seabird predation by river otters, we built a short survey for biologists to provide details about their observations in the field (Table S1, available in Supporting Information S1). This survey required biologists to report the seabird colony islands where they observed river otter activity, the species known to be depredated by river otters, and the form of evidence collected (scat, tracks, prey remains, direct visual observation, etc.). We did not require biologists to report any quantitative metric of predation impacts. We distributed this survey via professional society listservs (e.g., the Pacific Seabird Group) and direct individualized solicitation, using the latter option in a targeted manner to ensure broad geographic coverage across the species range of river otters. Many biologists we contacted also provided the survey to other individuals within their personal and professional networks, thereby extending our reach. We also collected references from published journal articles, white papers, and internal agency documents, although this was a non‐systematic search that often resulted from suggestions made by biologists. While we received many reports of predation on rafting and foraging seabirds in coastal waters, we restricted the scope of this paper to solely explore on‐land predation by river otters at nesting colonies.

Differences in overall predation frequency between seabird species are likely to be driven by distribution and abundance, rather than preferential selection by river otters. To more accurately capture true prey selection, we used a logistic modeling approach that incorporated the presence and abundance of nesting seabirds at colony islands where river otters are known to be present. For each colony island with reports of river otter activity, we located presence and abundance data for all nesting seabird species. These data were collated from many disparate sources, including published journal articles, government white papers, internal reports from environmental nonprofits, and graduate theses. It should be noted that many estimates of seabird abundance for these islands were more than two decades old, but we used the newest available data wherever possible. Each seabird species present on an individual island was included as a record in our dataset, and the observation (or lack thereof) of river otter predation on that species was modeled as the outcome. We converted species abundance numbers to proportional abundance for each colony island, used an empirical logit plot to assess the need to transform these data, and included this variable as an a priori fixed effect in every model.

To evaluate our hypotheses about river otter prey selection, we included covariates for seabird mass, nest type (surface, crevice, or earthen burrow), and nocturnal colony attendance. All seabird life history variables were sourced from the Birds of the World database (Billerman et al. 2022). No interactions between independent variables were included. We used an all‐subsets model selection approach (due to the small model set), with Akaike's Corrected Information Criterion (AICc) as our selection criteria to account for our limited sample size (Hurvich and Tsai 1989).

### 2.2 Factors Driving River Otter Occurrence at Seabird Colony Islands

To determine the variables associated with river otter occurrence at seabird colony islands, it was necessary to supplement our existing dataset with islands that have a confirmed *absence* of river otter activity. Thus, we sought to identify islands with established and active field research programs, such that there would be sufficient monitoring from biologists to detect any ongoing river otter activity. We reviewed all primary seabird research papers published between 2008 and 2023, and we recorded each instance of a seabird colony where field research occurred. Islands within the United States and Canada that were mentioned in at least three unique publications were selected for our dataset. Seabird colonies located in Nunavut, the Aleutian Islands, and the Pribilof Islands were considered out of the species range for river otters and were excluded from consideration.

We used paper authorship, professional networks, and word‐of‐mouth to locate biologists with field experience working on each of these islands. We then sent each of these authors a short, standardized survey which included questions about island characteristics, nesting seabird species, and river otter activity (Table S2). We also asked seabird biologists to report the number of years and weeks per year that they spent conducting field research on the island to determine whether detections of river otter activity were biased by monitoring intensity.

A final dataset was then constructed by merging the results from this targeted survey effort with the previously collected records. Each data point corresponded to a single seabird colony island, with the outcome variable indicating whether river otters had ever been observed there. Due to our mixed sampling approach, our reported outcomes are positively biased towards the overrepresentation of river otter activity, and this dataset cannot be used to estimate the exact probability of river otters occurring at seabird colony islands. Instead, we used logistic regression to assess the effects of different covariates on the *relative* likelihood of river otter occurrence across different islands. We considered a small set of potential covariates due to our limited sample size, including the presence of burrow‐nesting seabird species, distance to shore, island size, and the presence of persistent freshwater. We also included a potential interaction between persistent freshwater and distance to shore, as freshwater may enable permanent residency by river otters, thereby reducing the relative energetic cost of swimming a far distance to access a seabird colony island. Island size and distance to shore (defined as the minimum length from the island to a land mass of larger than 1000 ha) were measured in Google Earth, while information about the presence of freshwater on each island was provided by local biologists and published site descriptions. All continuous variables were assessed for transformation using empirical logit plots prior to modeling. We again used an all‐subsets model selection approach with AICc as our selection criteria.

### 2.3 Exploring Bias in Knowledge Across Biologist Specializations

To examine trends in existing publications on this topic, we attempted to locate every possible mention of seabird predation by river otters in a published journal article. We searched several major databases using a set of predefined search phrases (Table S4). We assessed all resulting papers for reported evidence of seabird predation by river otters, then sought out additional records via cited literature. We reviewed and scored all papers on a variety of categories, including the background of authors, the type of reference, the specialization of the publishing journal, and the number of words dedicated to discussing seabird predation. References were categorized as targeted studies (peer‐reviewed manuscripts where river otter predation was an a priori focus), anecdotal mentions (peer‐reviewed manuscripts about seabird or river otter ecology where opportunistic observations of predation were briefly mentioned), or field notes (short, non‐peer‐reviewed articles detailing observations of river otter predation). Summary statistics and exploratory visualization were then used to reach qualitative conclusions about trends in publication on this topic.

To further parse out biases in knowledge about this topic due to biologist specialization, we constructed a survey for river otter biologists with several questions pertaining to their observations and perceptions of seabird predation (Table S3). To build a survey recipient list, we reviewed all published journal articles about river otters from the last 20 years; any individuals with authorship on three or more papers were selected to receive this survey via email. By qualitatively comparing trends in the responses of otter biologists to those of seabird biologists, we were able to evaluate our hypotheses about how knowledge on this subject is accrued and disseminated.

## 3 Results

### 3.1 Nesting Seabird Prey Selection by River Otters

**FIGURE 1:**
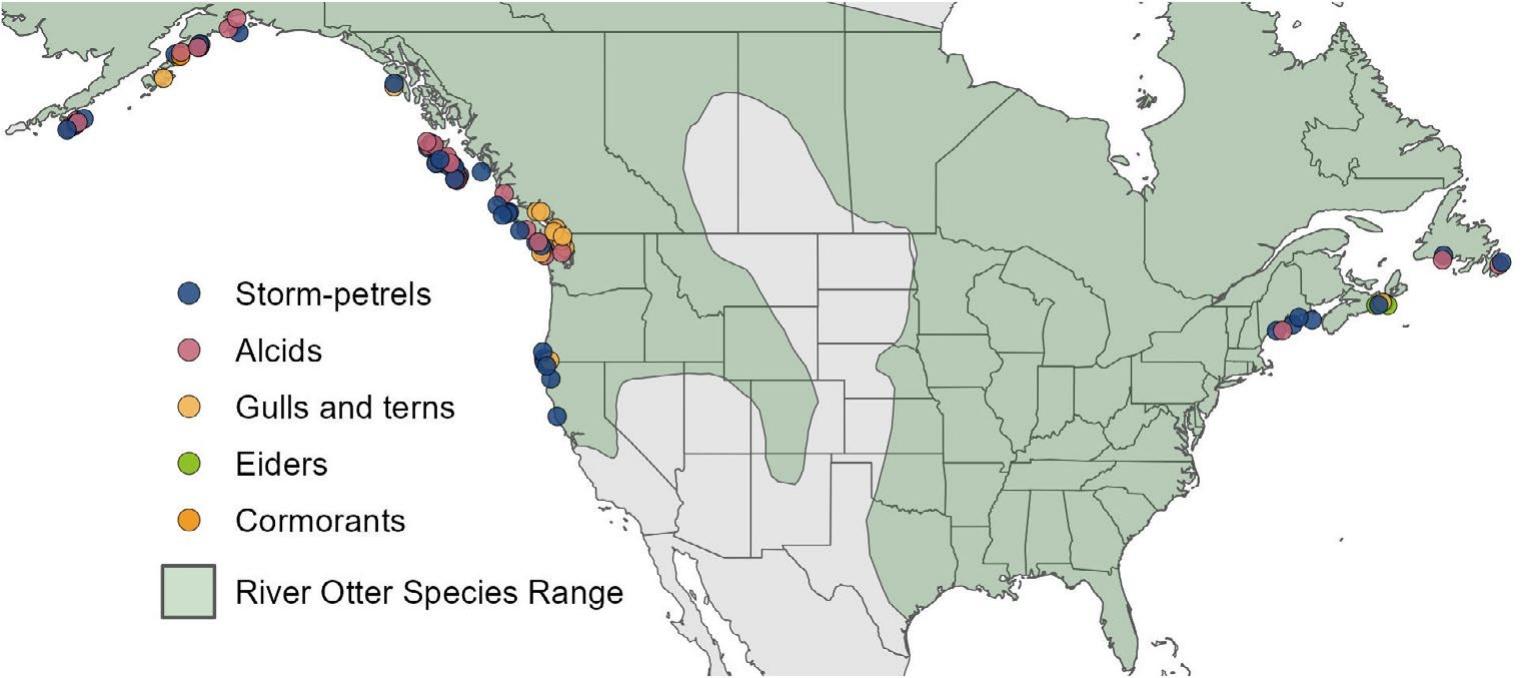
Locations of confirmed records of river otter predation at seabird colony islands throughout North America. Points are colored according to the family of seabird depredated. Point locations have been slightly jittered to avoid overlap. The species range of river otters has been overlaid in light green.

Across all survey efforts, we identified a total of 73 islands with confirmed records of seabird predation by river otters (Figure 1). We also collected information for 18 seabird colony islands with documented river otter activity but no observed seabird predation despite regular monitoring from biologists. These were included in our prey selection analysis, as river otters at these sites effectively selected against depredating any of the sympatric nesting seabirds. We were able to acquire full species abundance data for 79 of these islands, resulting in a final model dataset with a total sample size of 377 nesting records across 30 different seabird species. In naïve counts of predation occurrence, Leach's storm‐petrels (*Hydrobates leucorhous*) and fork‐tailed storm‐petrels (*Hydrobates furcatus*) were by far the most common, followed by glaucous‐winged gulls ( *Larus glaucescens* ), rhinoceros auklets ( *Cerorhinca monocerata* ), Cassin's auklets ( *Ptychoramphus aleuticus* ), and ancient murrelets ( *Synthliboramphus antiquus* ).

Our top performing model for seabird prey selection by river otters included log‐transformed proportional abundance, body mass, and nest type as meaningful predictors. A second model, which included a term for nocturnal provisioning, received some support (ΔAICc: 1.36), but the 95% confidence interval for this coefficient broadly overlapped zero (*β*: [−0.616, 1.526], *p* > 0.4), indicating that it was a largely uninformative predictor. In our top model, an increase in seabird relative body mass of 100 g corresponded to a 7.7% decrease in the odds of predation (95% CI: [−14.7%, −0.3%]). After accounting for abundance and body mass, river otters show a strong preference for burrow‐nesting species over all other types of nesters; with body mass and abundance held constant, burrow nesters have 2.8 times higher odds of being depredated than surface nesters, though the uncertainty around this odds ratio is broad (95% CI: [1.3×, 6.4×]). There is also non‐significant evidence that river otters may select for surface nesters over crevice nesters (95% odds ratio CI: [0.6×, 7.2×], *p* = 0.22).

**FIGURE 2:**
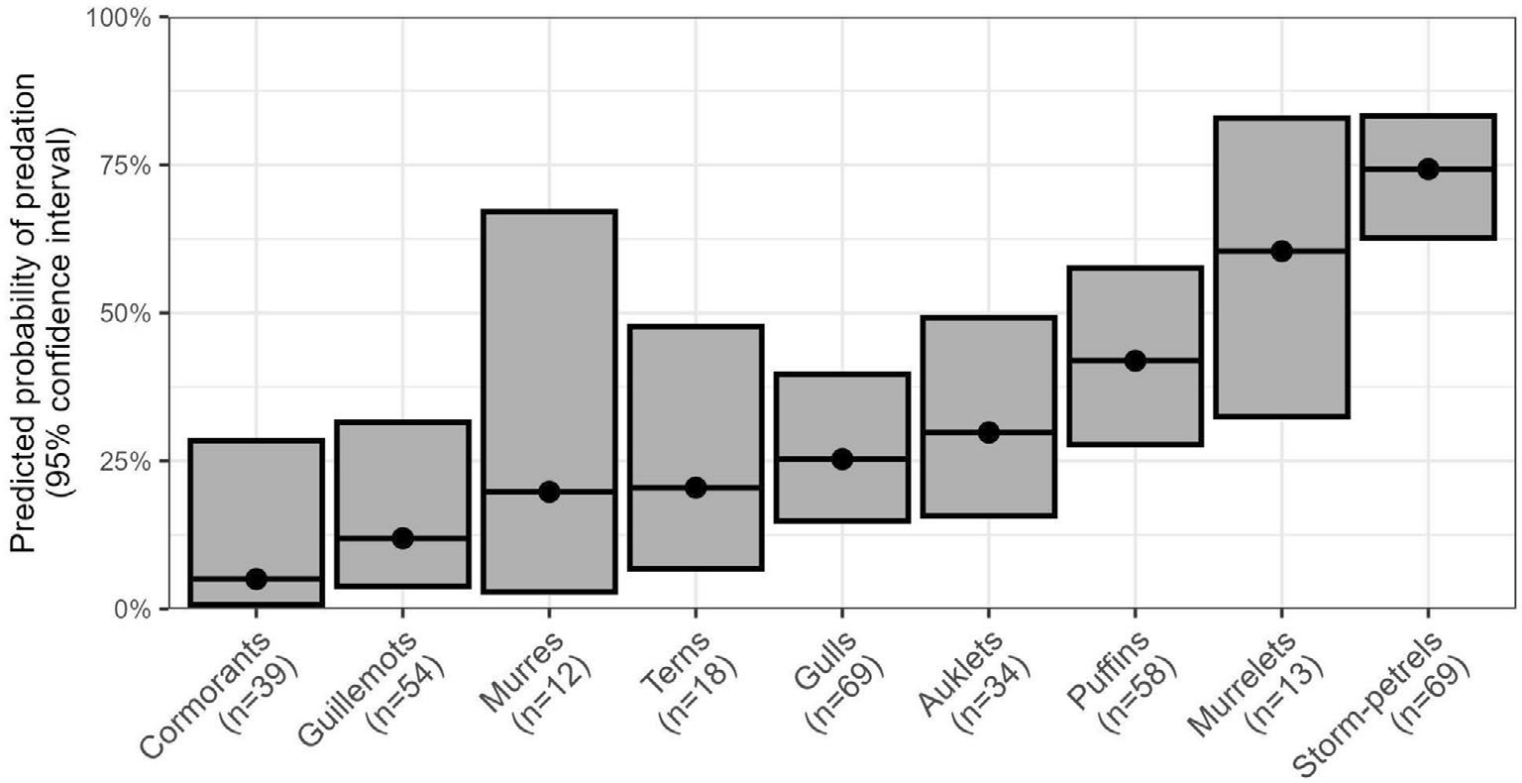
Predicted probability of predation for various taxonomic groups of seabirds, assuming the presence of river otters and that the nesting species makes up 25% of total colony abundance. Circular points represent the mean prediction, while the range of each bar represents a 95% confidence interval. This figure is intended for general comparative purposes, rather than true probabilistic prediction. Sample sizes refer to the number of records of nesting populations on islands with known river otter activity—not counts of cases where predation has occurred. Taxonomic groups with very low sample sizes (fulmars, eiders, pelicans, and skimmers) have been excluded. Groups refer to phylogenetic groupings, rather than common names—the “murre” group includes razorbills and the “puffin” group contains rhinoceros auklets.

After accounting for proportional abundance, the taxonomic groups which river otters show the highest selective preference for are storm‐petrels and murrelets (Figure 2)—both very small‐bodied nocturnally provisioning burrow nesters (marbled murrelets [ *Brachyramphus marmoratus* ] did not occur at any sites in our dataset). The non‐significant selective trend against crevice‐nesting species seems to be largely driven by pigeon and black guillemots ( *Cepphus columba* and *C. grylle* ), which experience extremely low rates of predation (Figure 2), especially given their relatively diminutive size. While horned puffins ( *Fratercula corniculata* ) and parakeet auklets ( *Aethia psittacula* ) are both crevice nesters, there were just seven instances of these species at islands in our database (compared to 54 instances of nesting guillemots).

### 3.2 Factors Driving River Otter Occurrence at Seabird Colony Islands

In addition to our set of 91 seabird colony islands with known river otter activity, we were able to collect confirmed negative records for 37 actively monitored islands throughout North America. Based on simple logistic regression, we saw no evidence of a relationship between monitoring intensity and detection of river otter activity at seabird islands with active research programs (*p* = 0.87). The top three performing models in our candidate set (cumulatively receiving 75% of AICc weight) all included meaningful effects for distance to shore and presence of burrow‐nesting seabirds. The second and third ranked models included terms for log‐transformed island area and persistent freshwater, respectively, but the 95% confidence intervals for these coefficients both widely overlapped zero.

**FIGURE 3:**
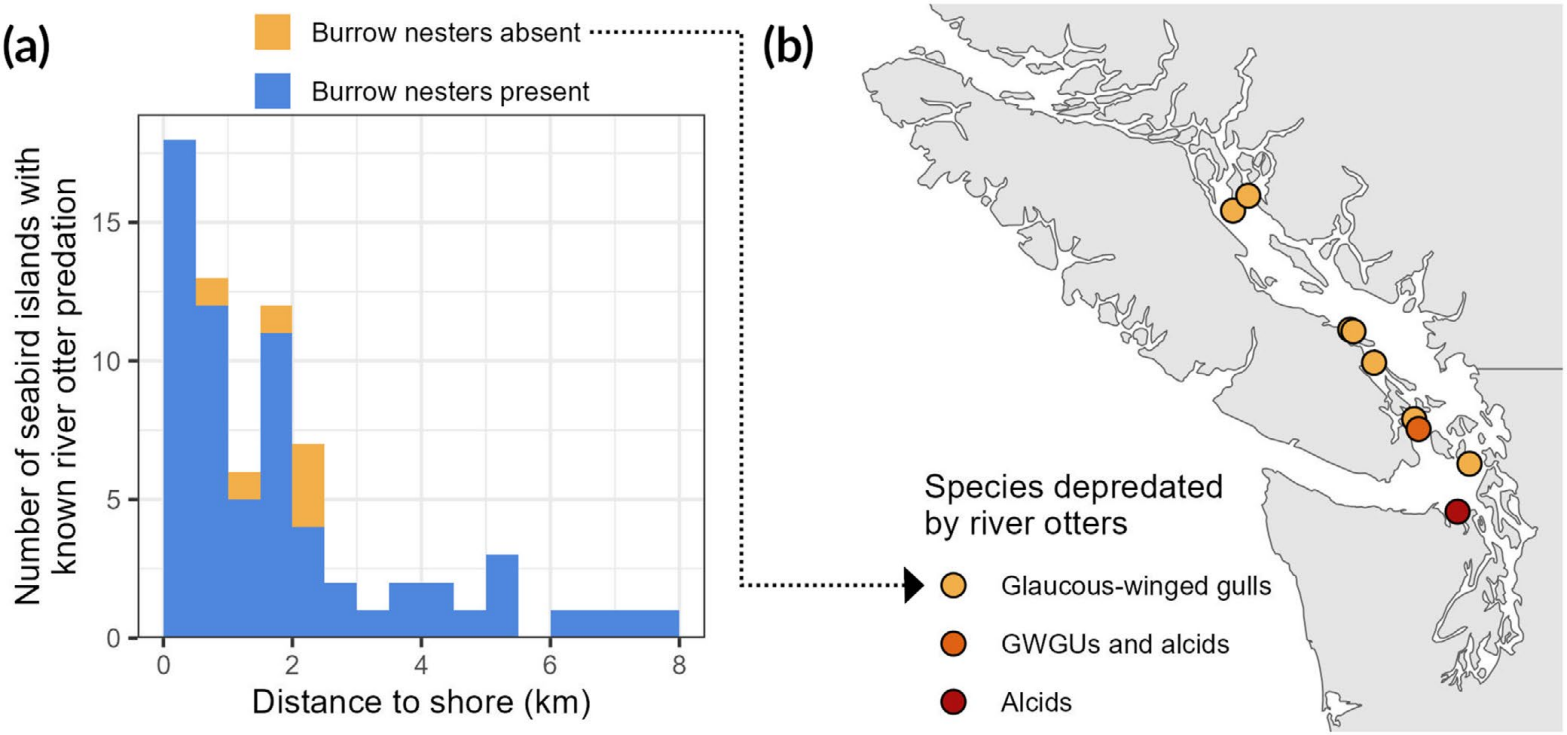
(a) Histogram of seabird colony islands with known river otter predation, binned by distance to shore. The colors of the bars represent whether burrow‐nesting seabirds were present at individual islands. (b) Map of seabird colony islands in the Salish Sea (British Columbia and Washington) with known river otter predation. Points represent individual colony islands, and the color of points represents the species depredated at each island (glaucous‐winged gulls vs. various species of alcid). The glaucous‐winged gull colonies in this region are the only known records of river otter predation at islands lacking burrow‐nesting seabirds. Seabird colony islands on the western coast of Vancouver Island are excluded from this plot.

Our top performing model predicted that the odds of river otter occurrence decline by 19.0% for each additional kilometer from shore (95% CI: [−32.6%, −7.8%], *p* < 0.01), and 69% of our records of river otter activity came from islands less than two kilometers from shore (Figure 3a). Despite this strong negative relationship between distance and predation risk, our dataset also includes multiple records of river otters making open‐ocean swims of up to eight kilometers to access seabird colony islands (Figure 3a). The presence of burrow‐nesting species was very strongly associated with river otter occurrence; our top model predicted that the odds of river otter activity are 19.7× higher for islands with burrow‐nesting species (95% odds ratio CI: [7.4×, 60.8×], (*p* < 0.001)), though effect sizes estimated from such a highly skewed sample should be interpreted with caution. River otter *predation* at islands without burrow‐nesting species (as opposed to just occurrence) was even more rare. We collected just 6 records of river otters hunting seabirds at colonies lacking burrow nesters, with all of these observations from river otters hunting glaucous‐winged gulls in the Salish Sea of British Columbia and Washington (Figure 3b). The limited amount of river otter activity at islands lacking burrow nesters cannot be explained solely by a shortage of small‐bodied seabirds, as many of these islands had sizable tern colonies (> 1000 pairs).

Seabird predation by river otters appears to be most common in the North Pacific (41% of our records are from British Columbia), but this behavior occurs throughout the Pacific coast and in the northern Atlantic (Figure 1). Notably, we were not able to identify a single instance of nesting seabird predation by river otters south of Maine on the Atlantic Coast or anywhere along the coast of the Gulf of Mexico. To ensure that this absence of predation records was not due to geographic bias in our survey distribution channels, we directly reached out to over twenty state, federal, academic, and nonprofit biologists working throughout this region. Many of these biologists had decades of field experience working with nesting seabirds, and not a single individual we spoke with had ever observed river otter predation in these areas. We did collect multiple records of river otter activity at seabird colony islands in both Massachusetts and South Carolina, but predation was not observed at any of these sites. It should be noted that when islands from the southern Atlantic Coast and the Gulf Coast were removed from our dataset, the top performing predictor variables for river otter occurrence remained the same (although coefficient values shifted somewhat).

### 3.3 Common Patterns in River Otter Predatory Behavior

In addition to information related to our targeted research questions, a number of patterns emerged in biologist survey responses that can provide some further insight into the predatory behavior of river otters at seabird colony islands. Because the quality of observations varied between sites, we cannot enumerate exactly how common certain behaviors were, but these anecdotal pieces of information provide a useful place for further discussion and exploration of the subject.

While river otter predation of burrow‐nesting species was reported to be fairly common, the specific mode of predation varied. In some cases, river otters excavated seabird burrows to access the nest chamber. This behavior was reported at storm‐petrel colonies in Alaska, British Columbia, Nova Scotia, and Oregon, and it seemed to be the primary mode of predation used by river otters at some islands. Despite accessing the nest chamber, river otters often seemed to actively avoid eating storm‐petrel eggs, instead opting to consume just chicks and adults (e.g., Quinlan 1983). At many other storm‐petrel colonies where river otter predation was fairly well observed, burrow excavation never occurred, and river otters exclusively killed adult birds outside of their burrows. For some islands with long‐term monitoring programs, the prevalence of burrow excavation varied dramatically between years despite the continued presence of river otters. At one site in British Columbia, river otters regularly entered artificial nest boxes to depredate pigeon guillemots, but this was effectively prevented by later reducing the diameter of nest box entrances. When hunting larger surface‐nesting species such as gulls, predation of both chicks and eggs seems to be relatively common (see Rodway et al. 2024 for examples), despite previous reports that river otters avoid consuming bird eggs (Hansen 2003).

River otter predation of seabirds primarily seems to be from seasonally transient visits to colony islands rather than permanent residence at these sites. River otters were present year‐round at just eight of 26 different islands where biologists reported knowledge of river otter residence patterns. In most cases where river otters were not living full‐time on the island, however, river otters made repeated visits to the colony throughout the breeding season to depredate seabirds; one‐off instances of predation seem to be quite rare. At many of these sites, river otters were reported to consistently use specific access paths onto the islands and developed well‐established trails. These trails often pass over remarkably difficult topography; at sites in Oregon and British Columbia, river otters regularly travel up > 50 m rocky cliffs with over a 150% grade to access seabird nesting areas.

River otters were not the only active seabird predators at most sites where they were observed. Co‐occurrence of river otters and American mink (*Neogale vison*) at seabird colonies seems to be a relatively common phenomenon. We gathered records of this overlap for sites in Alaska, British Columbia, Oregon, Newfoundland, Nova Scotia, and Maine—with many seabird biologists reporting that mink were a more important management issue than river otters. Biologists from both Rhode Island and Georgia reported that while river otters never depredate nesting seabirds despite their presence in the area, mink are active predators at these sites. Anecdotal evidence suggests that the ability or willingness of mink to swim far distances to access seabird colony islands may be more limited, as these reports of co‐occurrence between the two mustelid species were all from nearshore islands. Seabird predation by invasive American mink has received extensive attention in Europe (Craik 1997; Banks et al. 2008), but this has not been the subject of a comprehensive review in North America.

### 3.4 Exploring Bias in Knowledge Across Biologist Specializations

**FIGURE 4:**
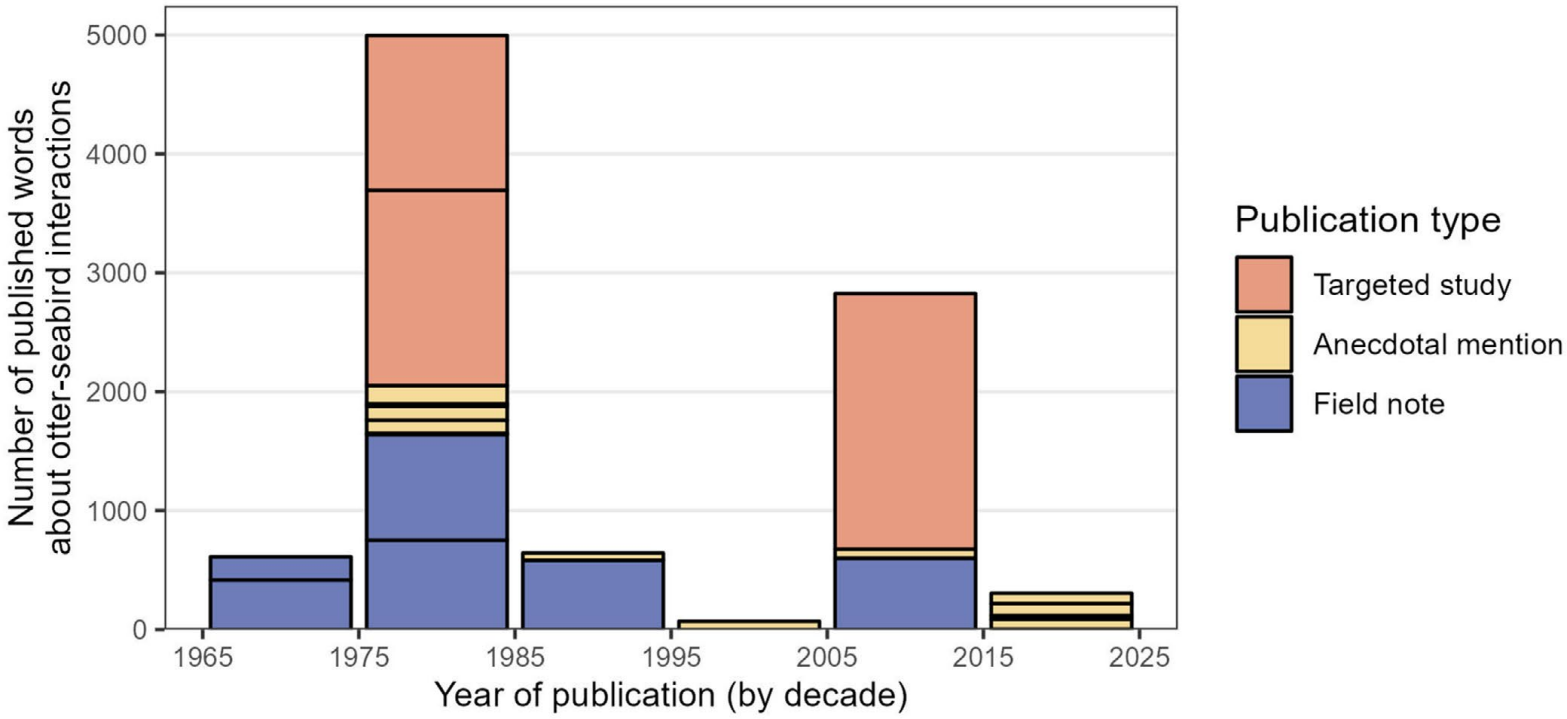
Bar chart of publications mentioning seabird predation by river otters, aggregated by decade. Individual cells represent a single publication, with the height of the cell representing how many words about the topic were included in the publication. Cells are colored according to the type of publication.

We were able to locate 22 total publications from peer‐reviewed journals which included direct mention of seabird predation by river otters (Table S5). Thirteen of these were secondary anecdotal mentions (often just a single sentence) in seabird population studies, six were “field note” style publications, and in just three instances was river otter predation of nesting seabirds an intentional subject of primary field research (Verbeek and Morgan 1978; Quinlan 1983; Carter et al. 2012). While many of these records were published in the last twenty years, the progressive disappearance of “field note” style publications in journals has resulted in a clear decline in the amount of writing on this subject (Figure 4). There was also a notable skew in the specialization of those publishing about this subject: just four of these articles had a mammologist in the author list. In the two cases where papers had a mammologist lead author, seabirds were only mentioned as a rare prey item in river otter scat, with a total of 24 words written about the subject between the two papers. Additionally, nine of these references came from ornithological journals, while another nine came from regionally restricted natural history journals.

We distributed surveys to 24 river otter biologists throughout North America, and we received responses from 16 of these biologists, who collectively have authorship on 92 unique publications about river otter ecology. Across this entire survey group, not a single river otter biologist had observed evidence of seabird predation in the field, and just five biologists reported any familiarity with secondhand reports of this behavior. Despite this absence of reported observations, river otter biologists generally agreed with the notion that seabird predation would not be dramatically outside their expectations, despite river otters' typical niche as aquatic predators. In their comments, six different biologists made reference to river otter predation on rafting and nesting waterfowl, and these biologists suggested that such behavior would likely translate to a coastal context. There appears to be a lack of consensus about the ability and willingness of river otters to make long‐distance swims in coastal environments; four biologists suggested that river otters would be unable or unwilling to swim more than 500 m to access a seabird colony island, while five other biologists predicted (remarkably accurately) that the upper threshold for this distance would be between five and ten kilometers.

## 4 Discussion

On‐land predation of nesting seabirds by river otters is neither rare nor exceptional. Across this study, we collected instances of river otters hunting eighteen different species of nesting seabirds along both coasts of North America. River otters demonstrate an adaptable ability to hunt a variety of seabird taxa with distinct morphologies and behavioral tendencies. Despite this dietary flexibility, river otters preferentially select for smaller‐bodied burrow‐nesting species. This result initially appears to contrast with studies of river otter foraging strategies in their primary freshwater habitats, where river otters have been shown to consistently select for larger‐bodied fish within individual taxa (Hansen 2003; Cote et al. 2008). In these habitats, however, the primary determinant of prey selection is almost always movement speed, with river otters selecting for slower species that are easier to capture and restrain (Ryder 1955; Day et al. 2015). In the case of highly mobile avian prey that pose an imminent flight risk, this is likely what drives the selection for smaller species—river otters seem more likely to take smaller prey that they can immediately kill or effectively restrain within their jaws. While successfully killing large seabirds on land may be challenging for river otters, we received many anecdotal reports of river otters hunting much larger species on the water, including murres, cormorants, and pelicans. In these cases, river otters swam underwater and attacked seabirds from below, restraining their legs in their jaws and drowning them before returning to shore to consume the carcass—a very similar strategy that has been observed on freshwater lakes where river otters hunt waterfowl (Reid et al. 1994; Price and Aries 2007). It seems that river otters expand their avian prey niche when hunting in aquatic environments, and the impacts of this variety of seabird predation may merit additional targeted research.

Interpreting river otter preference for burrow‐nesting species is complex, as this tendency does not correspond to a single foraging technique. While adult river otters can directly enter the burrows of larger bodied species (the burrows of North American puffin species average roughly 15 cm in diameter [Lowther et al. 2020], and river otters can squeeze through openings as tight as 10 cm [Reed‐Smith et al. 2008; Malzahn et al. 2020]), they will excavate the earthen burrows of smaller species (storm‐petrels, ancient murrelets, and Cassin's auklets) to access nest chambers. In many cases, however, biologists reported high rates of predation on storm‐petrels with limited or no evidence of burrow excavation—suggesting that river otters often hunt adult birds on the ground outside of burrows. This variability suggests that river otter preference for burrow nesters may be due to a difference in prey response rather than a specific predation tactic. While surface‐nesting species such as gulls and terns often exhibit alarm calls, mobbing, and fleeing in response to terrestrial predators (Clode et al. 2000), burrow‐nesting species have not been shown to demonstrate this type of coordinated group response to terrestrial predators. Collective vigilance is likely further limited during incubation when adults remain in their burrows for much of the day, and adults are unlikely to collectively flush from their burrows in response to a single predation attempt. This could allow for sustained foraging on burrow‐nesting species, whereas making repeated kills on surface nesters during a single hunting bout seems more unlikely. Prey response may also partially explain the lack of preference for nocturnally provisioning seabirds—if predator vigilance is reduced in diurnal species during the night, hunting success rates for nocturnally hunting river otters would be comparatively increased.

The apparent (though non‐significant) preference against hunting guillemots (Figure 2) is likely due to nest location and habitat, rather than restrictive access to crevice nests. While river otters would be unable to excavate crevice nests in the same manner that they do for earthen burrows, pigeon guillemots and black guillemots have average minimum cavity diameters of 15–20 cm (Cairns 1980; Oakley 1981), suggesting that most nests do not effectively exclude river otters. Locomotion and foraging may be more challenging in boulder fields and rocky scree than in flat vegetated areas, and guillemots at many islands will congregate on steep cliffs that may be fully inaccessible to river otters (Oakley 1981). This latter component may also explain the apparent selective preference against murres compared to other surface nesters (Figure 2), as these species often (though not exclusively) nest on precipitous cliff edges. Some anecdotal records report river otters scaling remarkably steep cliffs to access nesting areas, but it is logical that these predators would concentrate their foraging efforts on seabirds inhabiting flat areas of colony islands.

While river otters will take larger‐bodied surface‐nesting species, the anecdotal reports we collected suggest that this is primarily an opportunistic behavior exhibited at islands with an abundance of preferred prey. It appears that while gulls, terns, and murres may be suitable prey, it is not possible for river otters in most areas to become efficient enough hunters of surface nesters for them to become a primary prey item. If we were able to repeat the modeling done in this paper with quantified predation rates as the response variable (rather than a simple binary outcome), we hypothesize that the effect of nesting strategy would be even stronger. The Salish Sea is the only known location in the world where river otters have been consistently observed to visit islands to exclusively hunt a surface‐nesting species (glaucous‐winged gulls), and there have been local reports of this behavior for at least the last 60 years (Kennedy 1968; Rodway et al. 2024). This suggests that specialized seabird predation can be a locally learned and socially transmitted behavior, enabling certain populations to use an alternative prey resource. A similar localized behavioral adaptation in river otters has also occurred with non‐nesting brown pelicans along the Point Reyes National Seashore in California, where river otters have been regularly observed to kill rafting pelicans by drowning since at least 2006 (Carroll and Isadore 2024). Predation of brown pelicans has not been documented at any other sites in North America, providing further evidence that specialized seabird predation can occur as a behavioral innovation which is consistently exhibited by specific river otter subpopulations.

Our modeling results suggest that the presence of burrow‐nesting species is the single most powerful draw of river otters to seabird colony islands. Distance to shore is another very strong risk factor, and the true magnitude of this distance effect may be masked by the fact that seabirds could be actively selecting against nesting on islands that are very close to shore (within 2 km) due to the high threat of predation by native terrestrial mammals. Regardless of this trend, river otters show a willingness to make a high energetic investment to access foraging opportunities on seabird colony islands. River otters have a high basal metabolism relative to similarly sized mammals (Kruuk et al. 1994; Ben‐David et al. 2000), and thermo‐regulation in water is their primary source of energy expenditure while foraging (rather than locomotion) (Kruuk et al. 1997; Pfeiffer and Culik 1998). Thus, river otters must achieve a very high prey capture rate in cold marine conditions for energetic gains to offset the cost of thermoregulation (Kruuk 2006). To forego any attempt at aquatic foraging while swimming to a seabird colony island represents a major upfront investment. In ideal flatwater conditions, river otters can reach a movement speed of roughly 1.5 m/s (Fish 1994); it follows, therefore, that accessing many of the further afield islands (> 5 km) in our database would require a minimum of an hour of active swimming. River otters have been shown to use more terrestrial food sources during the winter to avoid the cost of cold‐water thermoregulation (Kruuk 2006), but the nesting season for seabirds in North America generally spans from March to August, when freshwater foraging should be relatively efficient. With all this taken into consideration, depredation of burrowing seabirds must provide a substantial energetic reward to offset the energetic cost of accessing far offshore islands.

Our modeling did not show any convincing evidence that island size or the presence of persistent freshwater increase the likelihood of river otters accessing a seabird colony island. Both of these hypotheses were made on the assumption that these factors would increase the suitability of an island for long‐term residence by river otters. The majority of our records indicate, however, that river otters tend to be transitory visitors to seabird colony islands. At one well‐studied nearshore seabird colony in southern Oregon, river otters swam to the island on a nightly basis to hunt storm‐petrels for a few hours before returning to the mainland (L. Stuntz unpublished data)—and many anecdotal reports we received describe a similar pattern. Dense colonies of burrow‐nesting seabirds offer a valuable enough foraging resource for river otters to repeatedly utilize them by swimming out from the mainland. It superficially appears that permanent or extended seasonal residence was more common on islands with sources of persistent freshwater, but we do not have sufficient data on river otter residence patterns to quantitatively assess this.

This dataset revealed an exceptionally robust geographical trend—river otter predation of seabirds appears to be non‐existent south of Maine on the Atlantic coast. This may be partially due to a lower density of both seabird colonies and coastal river otters in the region (compared to the Pacific coast and northern Atlantic), but this seems to be an insufficient explanation for the complete absence of any observed seabird predation. Instead, we hypothesize that this stark geographic split in the occurrence of predation is primarily the result of species assemblages at seabird islands on the Atlantic coast. The only burrow‐nesting species with colonies on the Atlantic coast are Leach's storm‐petrels and Atlantic puffins, and the most southern major colony of either of these species is located in St. George, Maine (Lowther et al. 2020; Pollet et al. 2021). These preferred prey species likely have a higher capture rate and require less energy to hunt, making it more likely for river otters to behaviorally adapt to include seabirds in their diet. While river otters will opportunistically take surface‐nesting seabirds in areas with dense burrow‐nesting colonies, it appears that the species assemblages south of Maine do not offer a valuable enough food resource for river otters to specialize as seabird predators.

Almost the entirety of existing knowledge on seabird predation by river otters seems to lie with seabird biologists; there are no notable publications on the subject by river otter biologists, and we did not obtain any new records of seabird predation from the river otter biologists we interviewed. In general, publications mentioning this behavior are sparse, declining in frequency, and difficult to opportunistically discover. Most journal articles mentioning seabird predation by river otters were from specialist or local journals, and very few articles mention the subject in their abstract. Seabird biologists seem to observe predation by river otters fairly frequently, but they very rarely study river otter behavior in a targeted manner or include their observations in publications. Through the course of this project, we spoke with over fifty active seabird biologists who have documented seabird predation by river otters, but there have been just 306 words published on the subject in the last decade. River otter biologists that we surveyed anticipated the ability of river otters to adapt to hunt seabirds in a coastal context, but they generally seemed to underestimate how valuable a foraging opportunity a densely nested seabird colony represents. We are aware of no previously published studies on river otter diets where birds (of any taxa) were the primary prey item, but through this project, we collected dozens of instances where river otters seasonally specialized to hunt seabirds instead of aquatic prey. Seabird colonies represent a habitat at the very edge of a river otter's niche, and they provide a unique insight into the extreme adaptability of this generalist predator. The role of terrestrial mammals in coastal systems has generally been understudied (Carlton and Hodder 2003), and the anecdotal knowledge of seabird biologists on a suite of native terrestrial species remains an underexplored scientific resource.

## 5 Management Implications

River otters have been underacknowledged as a native predator of nesting seabirds throughout North America. There are very few references to this species interaction in the literature (particularly in wildlife management journals), yet it is a fairly common occurrence in areas with large populations of burrow‐nesting seabirds. That being said, very few seabird biologists who had direct experience with river otter predation suggested that river otters represented a serious threat to the future of the colonies they study. River otters coexist with seabirds at many sites in North America without exhibiting any predatory tendencies, instead using the coasts of seabird islands as foraging grounds for their typical marine prey (Rodway et al. 2020). We are aware of just one island in North America where there is compelling evidence that predation by river otters resulted in long‐term colony abandonment (Carter et al. 2012). Regardless, wildlife management professionals working in coastal areas should be aware of the threat which river otters may pose to certain seabird species. Due to the nocturnal behavior of otters and their typically transient appearance at islands, diagnosing them as a source of predation can be difficult—particularly when islands are only visited for periodic censuses of seabird populations. We recommend awareness of this issue and enhancing surveillance of potential predation at nearshore islands with burrow‐nesting seabird populations, but our findings suggest that direct management is likely to be necessary only in very specific circumstances.

Perhaps more than anything, this project highlights the importance of drawing on the knowledge of taxa‐specific experts to understand the behavior of certain species at the edge of their typical niche. The management of river otters in the United States typically falls under the purview of furbearer biologists working for state wildlife agencies, but our survey work suggests that this population has limited information about the behavior of river otters at coastal islands. These agencies are (sensibly) equipped to address management concerns within terrestrial habitat, but the issues associated with carnivores in the island environment are very different. Management of these cases should be approached as a collaboration between experts on both taxa, with an active acknowledgment that the unique context calls for flexibility in how we understand and respond to carnivore behavior.

## Author Contributions

**Luke Stuntz:** conceptualization (lead), data curation (lead), formal analysis (lead), methodology (lead), project administration (supporting), visualization (lead), writing – original draft (lead), writing – review and editing (equal). **Rachael A. Orben:** conceptualization (supporting), formal analysis (supporting), funding acquisition (lead), methodology (supporting), project administration (lead), supervision (lead), visualization (supporting), writing – original draft (supporting), writing – review and editing (equal).

## Conflicts of Interest

The authors declare no conflicts of interest.

## Supporting information


**Data S1:** Supporting Information.

## Data Availability

All the required data and code have been archived at Dryad and are available at https://doi.org/10.5061/dryad.gxd2547zv.
