# Supplementary material for: River Otter Predation of Nesting Seabirds Along the Coasts of North America
Source: Ecol Evol. 2025 Sep 13;15(9):e72118. doi: 10.1002/ece3.72118 (PMC12432334; doi:10.1002/ece3.72118)
Supplement: Supplementary file 1 — Data S1: Supporting Information. [file ECE3-15-e72118-s001.docx]

**SUPPORTING INFORMATION**

Stuntz, L., R. A. Orben. 2025. River otter predation of nesting seabirds along the coasts of North America. Ecology and Evolution.

**Table S1.** Survey intended to collect anecdotal records of seabird predation from biologists. Surveys were distributed via professional society listservs and direct solicitation. All surveys were administered online via Qualtrics.

| Survey question | Response options |
| --- | --- |
| What is your name? | *Open response* |
| What is the name of the seabird colony island where you observed predation of seabirds by river otters? | *Open response* |
| What state or province is this seabird colony located in? | *Open response* |
| Which seabird species have you (or your field team) observed to be depredated by river otters on this island? | *Open response* |
| What type of evidence for predation did you observe? Please check all that apply. | – Direct visual observation of predation  – Seabird remains in river otter scat  – Seabird remains around river otter trails, dens, or latrines  – Camera trap footage  – Circumstantial: occurrence of river otters at the island and subsequent colony decline/abandoment, but no direct evidence observed  – Other *(Open response)* |
| What other seabird species are known to nest on this island? Please list either AOU 4-letter codes or common names. | *Open response* |
| Do you have any other thoughts about river otter predation that you would like to share? Any specifics about field observations, speculation about their impacts, or anecdotal ideas are more than welcome. | *Open response* |

**Table S2.** Targeted survey sent directly to biologists with experience working on islands with long-term seabird monitoring programs. Arrows indicate conditional survey questions, which would only appear depending on the response to a previous question. All surveys were administered online via Qualtrics.

| Survey question | | | Response options |
| --- | --- | --- | --- |
| What is your name? | | | *Open response* |
| What is the name of the seabird colony island which you are completing this survey in reference to? | | | *Open response* |
| During what years did you or your field team work on this island (e.g., 2003 - 2015)? If you cannot recall exact dates, a rough estimate is fine. | | | *Open response* |
| On average, how many weeks per field season are/were you (or your field team) present on this island? | | | *Open response* |
| What seabird species are known to nest on this island? Please list either AOU 4-letter codes or common names. | | | *Open response* |
| To your knowledge, is there any source of freshwater on this island? | | | – Yes, there are persistent freshwater streams/springs on the island  – There are no persistent streams/springs on the island, but there are rainfed pools of freshwater that persist for at least two weeks per field season  – There are no persistent sources of freshwater on the island  – I do not recall/know whether any freshwater is present on the island |
| Have you (or your field team) ever observed river otters to be present on this island? | | | – Yes – No |
| 🡪 | To what extent are river otters resident on this island? | | – River otters rarely appear on the island, and only for short periods of time  – River otters frequently appear on the island, but only for short periods of time  – River otters are (or have been) seasonally resident on the island (continual presence for over a week)  – River otters are (or have been) resident on the island year-round  – I do not recall/know about the residency patterns of river otters on the island |
| 🡪 | Have you (or your field team) ever observed evidence of seabird predation by river otters on this island? This may include otter scat containing bird remains, partially consumed bird carcasses, camera trap photos, or direct observation. | | – Yes, we have observed evidence of seabird predation by river otters  – No, we have never observed evidence of seabird predation by river otters  – I do not recall whether seabird predation by river otters was ever observed |
|  | 🡪 | Which seabird species have you (or your field team) observed to be depredated by river otters on this island? Please list common names. | *Open response* |
|  | 🡪 | On a scale of 1 to 5, how serious of a threat do you believe that river otters are to the future persistence of seabird colonies on this island? A response of 1 would indicate that otters present effectively no threat to colony persistence, while a response of 5 would indicate that otters will very likely cause extirpation of at least one seabird species. | *Sliding scale from 1 through 5* |
| Do you have any other thoughts about river otter predation that you would like to share? Any specifics about field observations, speculation about their impacts, or anecdotal ideas are more than welcome. | | | *Open response* |

**Table S3.** Survey sent to published river otter biologists to collect information about their observations, knowledge, and beliefs about seabird predation. Arrows indicate conditional survey questions, which would only appear depending on the response to a previous question. All surveys were administered online via Qualtrics.

| Survey question | | Response options |
| --- | --- | --- |
| What is your name? | | *Open response* |
| Have you ever personally observed any evidence of seabird predation by river otters while working in the field? This may include otter scat containing seabird remains, partially consumed seabird carcasses, camera trap photos, or direct behavioral observation. | | *–* Yes  – No |
| 🡪 | Briefly describe your personal observations of seabird predation by river otters. Please make sure to include both the location of the observation and the seabird species depredated. | *Open response* |
| Are you familiar with any other instances of seabird predation by river otters which you did not personally observe? This could include anecdotal observations by professional colleagues, published scientific papers, or reports from local communities where you complete fieldwork. | | *–* Yes  – No |
| 🡪 | Briefly describe your secondhand knowledge of seabird predation by river otters. Please include the location of predation, the seabird species depredated, and how you heard about this incident. | *Open response* |
| In a hypothetical situation where river otters inhabited an island with an active seabird colony, how serious of a threat do you believe that the otters could present to the future persistence of the nesting colony? A response of 1 would indicate effectively no threat to colony persistence, while a response of 5 would indicate that otters could very likely cause local extirpation of seabirds. | | *Sliding scale from 1 through 5* |
| Roughly how far from shore (in miles) do you believe a seabird colony island would have to be to be effectively insulated from any risk of predation by river otters? Put another way, how far out in the ocean do you believe a river otter would swim to access foraging opportunities on a seabird island? | | *Open response* |
| Do you have any other thoughts about seabird depredation that you would like to share? Any specifics about field observations, speculation about their impacts, or hypothetical ideas are more than welcome. | | *Open response* |

**Table S4.** Search phrases used in a systematic review of published articles directly mentioning seabird predation by river otters. Abbreviations for article databases/repositories: Web of Science (WoS), Searchable Ornithological Research Archive (SORA), Google Scholar (GS).

| Databases searched | Search phrase |
| --- | --- |
| WoS, SORA, GS | otter* AND seabird* NOT trawl* |
| WoS, SORA, GS | otter* AND storm-petrel* NOT trawl* |
| WoS, SORA, GS | otter* AND petrel* NOT trawl* |
| WoS, SORA, GS | otter* AND alcid* NOT trawl* |
| WoS, SORA, GS | otter* AND auklet* NOT trawl* |
| WoS, SORA, GS | otter* AND murre* NOT trawl* |
| WoS, SORA, GS | otter* AND puffin* NOT trawl* |
| WoS, SORA, GS | otter* AND guillemot* NOT trawl* |
| WoS, SORA, GS | otter* AND gull* NOT trawl* |
| WoS, SORA, GS | otter* AND tern* NOT trawl* |
| WoS, SORA, GS | otter* AND cormorant* NOT trawl* |
| WoS, SORA, GS | (lontra* OR lutra*) AND seabird* |
| WoS, SORA, GS | (lontra* OR lutra*) AND storm-petrel* |
| WoS, SORA, GS | (lontra* OR lutra*) AND petrel* |
| WoS, SORA, GS | (lontra* OR lutra*) AND alcid* |
| WoS, SORA, GS | (lontra* OR lutra*) AND auklet* |
| WoS, SORA, GS | (lontra* OR lutra*) AND murre* |
| WoS, SORA, GS | (lontra* OR lutra*) AND puffin* |
| WoS, SORA, GS | (lontra* OR lutra*) AND guillemot * |
| WoS, SORA, GS | (lontra* OR lutra*) AND gull* |
| WoS, SORA, GS | (lontra* OR lutra*) AND tern* |
| WoS, SORA, GS | (lontra* OR lutra*) AND cormorant* |
| SORA | "river otter" |
| SORA | "lontra canadensis" OR “lutra canadensis” |
| WoS, GS | "river otter"* AND diet* AND coastal* |
| WoS, GS | "river otter*" AND island* |
| WoS, GS | (lontra* OR lutra*) AND diet* AND coastal* |
| WoS, GS | (lontra* OR lutra*) AND island* |

**Table S5.** Articles in peer-reviewed journals which included direct mention of seabird predation by river otters found during a systematic literature review.

| Article name | Authors | Journal name | Publication year |
| --- | --- | --- | --- |
| River otter feeding on glaucous-winged gull | Ken Kennedy | Blue Jay | 1968 |
| Predation on nesting gulls by a river otter in Washington State | James Hayward, Charlers Amlaner, Humphrey Gillett, John Stout | The Murrelet | 1975 |
| Distribution and abundance of marine birds and mammals along the south side of the Kenai Peninsula, Alaska | Edgar Bailey | The Murrelet | 1977 |
| Predation on nesting glaucous-winged gulls by river otter | Robert Foottit, Robert Butler | The Canadian Field Naturalist | 1977 |
| Breeding seabird distribution and abundance in the Shumagin Islands, Alaska | Edgar Bailey | The Murrelet | 1978 |
| River otter predation on glaucous-winged gulls on Mandarte Island, British Columbia | Nicolaas Verbeek, Joan Morgan | The Murrelet | 1978 |
| The breeding biology of the fork-tailed storm petrel (*Oceanodroma furcata*) | Dee Boersma, Nathaniel Wheelwright, Mark Nerini, Eugenia Wheelwright | The Auk | 1980 |
| Avian and river otter predation in a storm-petrel colony | Susan Quinlan | Journal of Wildlife Management | 1983 |
| Feeding habits of river otters in coastal southeastern Alaska | Douglas Larsen | Journal of Wildlife Management | 1984 |
| Food habits of the river otter *Lutra canadensis* in the marine environment of British Columbia | GB Stenson, GA Badgero, HD Fisher | Canadian Journal of Zoology | 1984 |
| River otter occurrence and predation on nesting marine birds in the Washington Islands Wilderness | Steven Speich, Robert Pitman | The Murrelet | 1984 |
| Comparison of nesting biology of fork-tailed and Leach's storm-petrels | Kees Vermeer, Kevin Devito, Leo Rankin | Colonial Waterbirds | 1988 |
| Apparent river otter predation at an Aleutian tern colony | David Cameron Duffy | Colonial Waterbirds | 1994 |
| The impact of raccoons *Procyon lotor* on breeding seabirds at Englefield Bay, Haida Gwaii, Canada | Anthony Gaston, Michelle Masselink | Bird Conservation International | 1997 |
| A river otter's *Lontra canadensis* capture of a double-crested cormorant *Phalacrocorax auritus* in British Columbia's Gulf Island waters | Michael Price, Claire Aries | The Canadian Field Naturalist | 2007 |
| Breeding of the ashy storm-petrel in central Mendocino County, California | Harry Carter, Michael Parker, Josh Koepke, Darrell Whitworth | Western Birds | 2012 |
| Historical colony status and recent extirpations of burrow-nesting seabirds at Seabird Rocks, British Columbia | Harry Carter, Alan Burger, Peter Clarkson, Yuri Zharikov, Michael Rodway, Spencer Sealy, Wayne Campbell, David Hatler | Wildlife Afield | 2012 |
| Breeding population decline and associations with nest site use of Leach’s Storm-Petrels on Kent Island, New Brunswick from 2001 to 2018 | Kyle d'Entremont, Laura Zitske, Alison Gladwell, Nathan Elliott, Robert Mauck, Robert Ronconi | Avian Conservation & Ecology | 2020 |
| Levels of predation at two Leach's storm-petrel *Hydrobates leucorhous* breeding colonies | Rielle Hoeg, Dave Shutler, Ingrid Pollet | Marine Ornithology | 2021 |
| Variation in diet and activity of river otters (*Lontra canadensis*) by season and aquatic community | Hilary Cosby, Micaela Szykman Gunther | Journal of Mammalogy | 2021 |
| Understanding widespread declines for common terns across inland North America: productivity estimates, causes of reproductive failure, and movement of common terns breeding in the large lakes of Manitoba | Jennifer Arnold, Stephen Oswald, Scott Wilson, Patricia Szczys | Avian Conservation & Ecology | 2022 |
| Apparent survival among adult Leach's storm-petrels *Hydrobates leucorhous* on a colony managed for predators in Nova Scotia, Canada | Sarah Gutowsky, Gregory Robertson, Anna Calvert, David Fifield, Robert Ronconi, Jennifer Rock | Marine Ornithology | 2023 |

**Literature Cited for Table S5**

Arnold, J. M., S. A. Oswald, S. Wilson, and P. Szczys. 2022. Understanding widespread declines for Common Terns across inland North America: productivity estimates, causes of reproductive failure, and movement of Common Terns breeding in the large lakes of Manitoba. Avian Conservation and Ecology 17.

Bailey, E. P. 1977. Distribution and abundance of marine birds and mammals along the south side of the Kenai Peninsula, Alaska. The Murrelet 58:58–72.

Bailey, E. P. 1978. Breeding seabird distribution and abundance in the Shumagin Islands, Alaska. The Murrelet 59:82–91.

Boersma, P., N. Wheelwright, and M. Nerini. 1980. The breeding biology of the fork-tailed storm-petrel (*Oceanodroma Furcata*). The Auk 97:268–282.

Carter, H. R., A. E. Burger, P. V Clarkson, Y. Zharikov, M. S. Rodway, S. G. Sealy, R. W. Campbell, and D. F. Hatler. 2012. Historical colony status and recent extirpations of burrow-nesting seabirds at Seabird Rocks, British Columbia. Wildlife Afield 9:13–48.

Carter, H. R., M. W. Parker, J. S. Koepke, and L. Darrell. 2015. Breeding of the ashy storm-petrel in central mendocino county, california. 49–65.

Cosby, H., and M. S. Gunther. 2021. Variation in diet and activity of river otters (*Lontra canadensis*) by season and aquatic community. Journal of Mammalogy 102:520–529.

D’entremont, K. J. N., L. M. Zitske, A. J. Gladwell, N. K. Elliott, R. A. Mauck, and R. A. Ronconi. 2020. Breeding population decline and associations with nest site use of Leach’s storm-petrels on Kent Island, New Brunswick from 2001 to 2018. Avian Conservation and Ecology 15:1–11.

Duffy, D. C. 1995. Apparent river otter predation at an Aleutian tern colony. Colonial Waterbirds 18:91–92.

Foottit, R. G., and R. W. Butler. 1977. Predation on nesting glaucous-winged gulls by river otter. The Canadian Field-Naturalist 91:189–190.

Gaston, A. J., and M. Masselink. 1997. The impact of raccoons *Procyon lotor* on breeding seabirds at Englefield Bay, Haida Gwaii, Canada. Bird Conservation International 7:35–51.

Gutowsky, S. E., G. J. Robertson, A. M. Calvert, D. A. Fifield, R. A. Ronconi, and J. C. Rock. 2023. Apparent survival among adult Leach’s storm-petrels *Hydrobates leucorhous* on a colony managed for predators in Nova Scotia, Canada. Marine Ornithology 51:65–72.

Hayward, J. L., C. J. Amlaner, W. H. Gillett, and J. F. Stout. 1975. Predation on nesting gulls by a river otter in Washington state. The Murrelet 56:9–10.

Hoeg, R., D. Shutler, and I. L. Pollet. 2021. Levels of predation at two Leach’s storm-petrel *Hydrobates leucorhous* breeding colonies. Marine Ornithology 49:119–125.

Kennedy, K. 1968. River otter feeding on glaucous-winged gull. The Blue Jay 26:109.

Larsen, D. N. 1984. Feeding habits of river otters in coastal southeastern Alaska. The Journal of Wildlife Management 48:1446–1452.

Price, M. H. H., and C. E. Aries. 2007. A river otter’s*, Lontra canadensis*, capture of a double-crested cormorant, *Phalacrocorax auritus*, in British Columbia’s Gulf Island Waters. Canadian Field Naturalist 121:325–326.

Quinlan, S. E. 1983. Avian and river otter predation in a storm-petrel colony. Journal of Wildlife Management 47:1036–1043.

Speich, S. M., and R. L. Pitman. 1984. River otter occurrence and predation on nesting marine birds in the Washington Islands Wilderness. The Murrelet 65:25–27.

Stenson, G. B., G. A. Badgero, and H. D. Fisher. 1984. Food habits of the river otter Lutra canadensis in the marine environment of British Columbia. Canadian Journal of Zoology 62:88–91.

Verbeek, N. A. M., and J. L. Morgan. 1978. River otter predation on glaucous-winged gulls on Mandarte Island, British Columbia. The Murrelet 59:92–95.

Vermeer, K., K. Devito, and L. Rankin. 1988. Comparison of nesting biology of fork-tailed and Leach’s storm-petrels. Colonial Waterbirds 11:46–57.
